# Magnesium Membrane Shield Technique for Alveolar Ridge Preservation: Step-by-Step Representative Case Report of Buccal Bone Wall Dehiscence with Clinical and Histological Evaluations

**DOI:** 10.3390/biomedicines12112537

**Published:** 2024-11-06

**Authors:** Marko Blašković, Ivana Butorac Prpić, Serhat Aslan, Dragana Gabrić, Dorotea Blašković, Olga Cvijanović Peloza, Marija Čandrlić, Željka Perić Kačarević

**Affiliations:** 1 Dental Clinic Dr. Blašković, Linićeva ulica 16, 51000 Rijeka, Croatia; marko_blaskovic@yahoo.com (M.B.); dorotea.mihanovic@gmail.com (D.B.); 2 Department of Oral Surgery, Faculty of Dental Medicine Rijeka, University of Rijeka, Krešmirova ulica 40/42, 51000 Rijeka, Croatia; 3 Department of Dental Medicine, Faculty of Dental Medicine and Health Osijek, J.J. Strossmayer University of Osijek, 31000 Osijek, Croatia; butoracivana88@gmail.com; 4 Department of Biomedical, Surgical and Dental Sciences, University of Milan, 20100 Milan, Italy; dr.serhataslan@gmail.com; 5 Department of Oral Surgery, School of Dental Medicine University of Zagreb, 10000 Zagreb, Croatia; dgabric@sfzg.hr; 6 Department of Dental Medicine, Clinical Hospital Centre Zagreb, 10000 Zagreb, Croatia; 7 Department of Anatomy, Faculty of Medicine, University of Rijeka, Braće Branchetta 20/1, 51000 Rijeka, Croatia; olga.cvijanovic@uniri.hr; 8 Department of Integrative Dental Medicine, Faculty of Dental Medicine and Health Osijek, J.J. Strossmayer University of Osijek, 31000 Osijek, Croatia; 9 Department of Anatomy, Histology, Embriology, Pathology Anatomy and Pathology Histology, Faculty of Dental Medicine and Health Osijek, J.J. Strossmayer University of Osijek, 31000 Osijek, Croatia; 10 Botiss Biomaterials GmbH, 15806 Zossen, Germany

**Keywords:** case report, guided tissue regeneration, magnesium, membrane, histology

## Abstract

Background/Objectives: Despite the increased use of new resorbable magnesium membranes, there are no reported cases or studies on the use of resorbable magnesium membranes in combination with bone grafts for alveolar ridge preservation (ARP) in cases with severe buccal bone wall dehiscence. This case report aimed to evaluate the effectiveness of the magnesium membrane shield technique in conjunction with bone grafting for ARP, assessing both clinical outcomes and histological bone regeneration. Methods: A 44-year-old female patient presented with a vertical fracture on tooth 24 (FDI Notation System) accompanied with complete destruction of the buccal bone wall. The treatment plan included tooth extraction, ARP using a combination of anorganic bovine bone and autologous bone grafting, and the application of a magnesium membrane as a shield to the pre-existing buccal wall. Six months post-procedure, a bone biopsy was taken from the implant site using a trephine bur. Results: Clinical and radiological evaluations six months after the procedure demonstrated sufficient bone volume for implant placement. Additionally, in the next three months, soft tissue conditioning with a provisional crown resulted in an aesthetically and functionally satisfactory outcome. Histological analysis of the bone biopsy revealed well-formed new bone in direct contact with residual biomaterial, with no signs of inflammation. Osteocytes were clearly visible within the newly formed bone matrix, indicating successful bone maturation. Active osteoblasts were observed along the bone-biomaterial interface, suggesting ongoing bone remodeling and integration. Additionally, histomorphometric evaluation revealed 47% newly formed bone, 32% soft tissue, and 19% residual biomaterial. Conclusions: This case demonstrates the potential of the magnesium shield technique as an ARP technique in cases with severe buccal wall dehiscence. The technique yielded satisfactory clinical outcomes and promoted successful bone regeneration, as confirmed by histological analysis.

## 1. Introduction

Natural teeth are superior to implant-prosthetic restorations due to their proprioceptive capabilities and ability to adapt to mechanical loads. However, in certain clinical situations, such as advanced periodontal disease, failed root canal treatment, vertical root fractures, or non-restorable teeth, extraction becomes necessary [1]. Following tooth removal, the socket undergoes a series of healing phases that result in the formation of new bone tissue, accompanied by changes in both the horizontal and vertical dimensions of the alveolar ridge [2,3]. These dimensional alterations present a challenge when transitioning from a natural tooth to a prosthetic restoration, as maintaining both aesthetics and function becomes more difficult due to the inevitable bone resorption [4,5,6].

Implant-prosthetic therapy has become one of the most popular options for replacing missing teeth due to its high predictability and favorable long-term survival rates, with success rates of 96.4% after 10 years [7,8] and 91.5% after 16 to 22 years of follow-up [9]. Correct three-dimensional implant placement is essential for achieving predictable, long-term, and aesthetically satisfying results [8]. Insufficient bone volume at the implant site can compromise ideal placement, leading to functional and aesthetic complications, as well as long-term instability of the peri-implant hard and soft tissues [8,10,11]. Additionally, in clinical evaluations before implant placement, it is crucial to assess the extent of the alveolar bone defect, which may present at various stages, including before tooth extraction, in situations such as vertical root fractures [12], during extraction, in cases of traumatic tooth removal [13], and after extraction, as a result of physiological bone remodeling, especially in patients with a thin bone biotype (bone thickness ≤ 1 mm) [14].

Horizontal and vertical bone loss after tooth extraction is particularly pronounced in the thin, tooth-dependent bundle bone that lines the socket. The loss of blood supply from the periodontal ligament triggers osteoclastic activity, causing approximately 2.2 mm of vertical bone loss on the buccal side, especially in mandibular premolars, while the thicker lingual bone experiences much less resorption [15,16].

To address this, alveolar ridge preservation (ARP) techniques were developed with the aim to reduce bone volume loss in both dimensions, thereby decreasing the need for simultaneous bone augmentation during implant placement [17]. A study by Araujo et al. [18] demonstrated that socket grafting with xenogenic bone graft enriched with 10% collagen can significantly reduce post-extraction bone loss. It is estimated that ARP techniques can preserve 85–90% of the initial bone volume after healing [19]. Additionally, systematic review by Bassir et al. [11] suggests that with ARP, reductions in bone loss of 1.86 mm horizontally and 1.36–1.55 mm vertically can be expected.

The magnesium membrane is a recently developed biomaterial designed to address the limitations of conventional membranes used in regenerative procedures. It maintains structural integrity during the critical healing phase, providing support while gradually resorbing over time, thereby eliminating the need for a secondary surgical procedure for removal. Moreover, the degradation products of the magnesium membrane are fully absorbed by the body without producing any harmful by-products [20]. Published papers have demonstrated the biological advantages of magnesium in promoting tissue vascularization and have provided insight into the mechanisms by which magnesium ions promote both angiogenesis and osseointegration [21,22]. Our group has already reported clinical cases showing the potential of magnesium membranes for bone regeneration, including their application for immediate alveolar bone regeneration, treatment of intrabony defects, sinus lift, and immediate and delayed implant placement [23,24,25]. However, there are no reports on the use of magnesium membranes in combination with bone grafts for alveolar ridge preservation in cases of extensive buccal wall dehiscence using an open healing approach. Therefore, in this paper, we aim to assess the clinical and histological outcomes of using a magnesium membrane in combination with bone grafting for ARP in a representative, step-by-step case of large buccal wall dehiscence.

## 2. Materials and Methods

### 2.1. Patient Description, Case History, and Ethical Considerations

This case report follows the CARE (CAse REports) guidelines, with the CARE checklist included in the Supplementary Materials [26]. Ethical approval was granted by the Ethical Committee of the Faculty of Dental Medicine and Health at the University J.J. Strossmayer of Osijek, Croatia, and the procedure was conducted in accordance with the Declaration of Helsinki (ethical approval: Class: 602-01/23-12/05, No. 2158/97-97-10-23-03). Prior to the treatment, the patient was thoroughly informed about the diagnostic procedures and treatment options. Following this discussion, written informed consent was obtained for the chosen treatment protocol and for publication of this case report.

**Figure 1:**
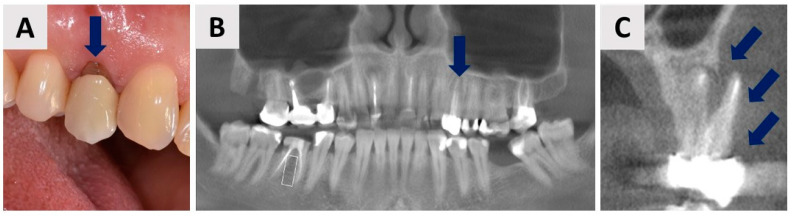
Initial clinical and radiological examinations. (**A**) Labial view of tooth 24 (FDI notation). (**B**) Panoramic CBCT section showing radiolucency at the apex of tooth 24 (blue arrow). (**C**) Coronal CBCT section revealing complete destruction of the buccal bone wall in the region of tooth 24. Blue arrows mark the area where the buccal bone wall previously existed.

The patient, a 44-year-old female, was in good general health, with no chronic medical conditions or medications. The patient was a non-smoker, had good oral hygiene (Full Mouth Plaque Score, FMPS = 10%), and demonstrated strong compliance with the recommended treatment. She presented with a desire for full-mouth aesthetic and functional rehabilitation due to perceived deficiencies in both the maxilla and mandible. Clinical examination of tooth 24 (FDI Dual Notation System) revealed gingival recession around the prosthetic crown and a vertical fracture line extending from the crown margin to the marginal gingiva. Cone beam computed tomography [(CBCT), Veraviewepocs 3D R100J, Morita, Osaka, Japan] confirmed previous endodontic treatment, a periapical lesion, and complete destruction of the buccal bone wall (Figure 1).

Given the diagnosis of a vertical root fracture, extraction of the tooth was necessary to prevent further complications. The treatment plan involved extraction, ARP, and subsequent dental implant placement after six months of healing. Additionally, for scientific purposes, the patient consented to a bone biopsy prior to implant placement, which would be performed using a trephine bur for histological analysis.

### 2.2. Planning the Magnesium Membrane Shield Technique Surgery and Materials Used

**Figure 2:**
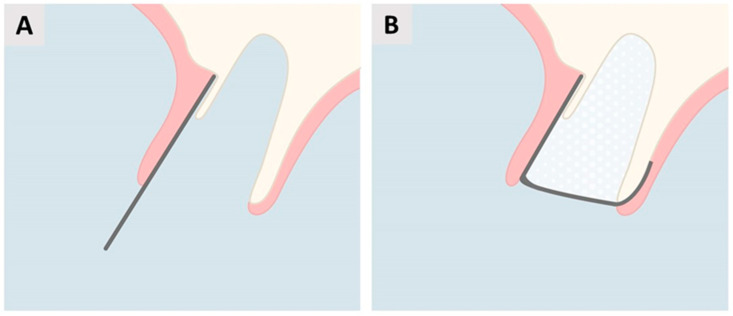
Schematic representation of the planned magnesium membrane shield technique for alveolar ridge preservation (ARP) following tooth extraction. (**A**) Placement of the magnesium membrane along the buccal wall beneath the gingiva. (**B**) The socket will be filled with mixture of anorganic bovine bone and autogenous bone. Then, the membrane will be folded over the socket and positioned on the palatal wall beneath the soft tissue.

Based on clinical and radiological examinations and confirmation by direct visual inspection following tooth extraction, the socket was classified as defect type ST3C according to Steigmann et al. [27], characterized by a buccal bone wall dehiscence exceeding two-thirds of the wall height. Figure 2 illustrates the schematically planned procedure to be performed following tooth extraction.

**Figure 3:**
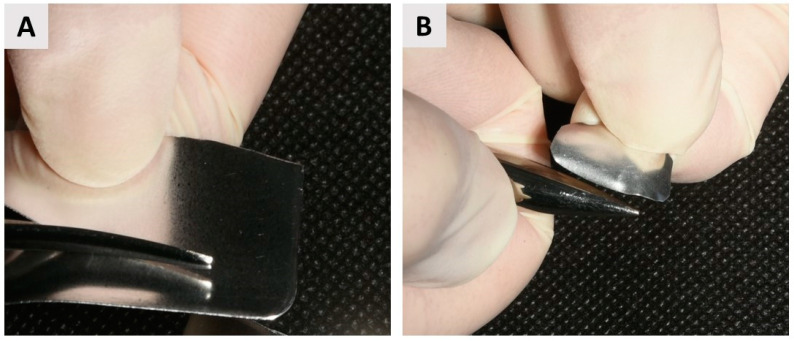
Magnesium membrane cutting and shaping. (**A**) Cutting the magnesium membrane into the appropriate shape using NOVAMag^®^ scissors. The membrane should be sized to extend beyond the entire buccal defect, covering an additional 3 mm of intact bone in the apical, mesial, and distal directions to ensure adequate stabilization. Additionally, the membrane should extend over the crestal portion of the alveolus, which will remain uncovered by the flap, to minimize the risk of graft displacement, bacterial colonization, and contamination associated with oral hygiene challenges. It should also cover up to 3 mm of the palatal bone wall to enhance stabilization and ensure secure positioning throughout the healing process; (**B**) Smoothing the sharp edges of the membrane with the NOVAMag^®^ sculptor.

Following tooth extraction and thorough debridement, a small intrasulcular incision was made to allow elevation of a flap extending to the apex of the former root, facilitating access to the area with a buccal bone wall defect. This approach allows adequate exposure to enable precise insertion of the magnesium membrane (NOVAMag^®^ membrane, botiss biomaterials GmbH, Berlin, Germany) (Figure 3). Just before placing it into the defect, the membrane was shaped to 8 × 12 mm using specialized NOVAMag^®^ scissors (Carl Martin GmbH, Solingen, Germany) (Figure 3A), and the edges were smoothed with the NOVAMag^®^ sculptor (Carl Martin GmbH, Solingen, Germany) (Figure 3B) to prevent perforation of the overlying soft tissue.

The membrane was positioned beneath the periosteum, covering the entire defect area and extending an additional 3 mm onto the adjacent intact bone in the apical, mesial, and distal directions to ensure optimal stabilization and coverage (Figure 2A). The socket was then be filled with a bone graft material—a mixture containing 60% autogenous bone that was harvested from the maxillary tuberosity in the same quadrant and 40% anorganic bovine xenograft (cerabone^®^, botiss biomaterials GmbH, Zossen, Germany). Then, the membrane was bent and rolled over the crestal part of the ridge and tucked below the palatal flap (Figure 2B). This approach maintains the membrane’s placement while allowing for open healing, as the membrane is left exposed for healing by secondary intention (per secundam).

### 2.3. Tooth Extraction and Alveolar Ridge Preservation with the Magnesium Membrane Shield Technique

**Figure 4:**
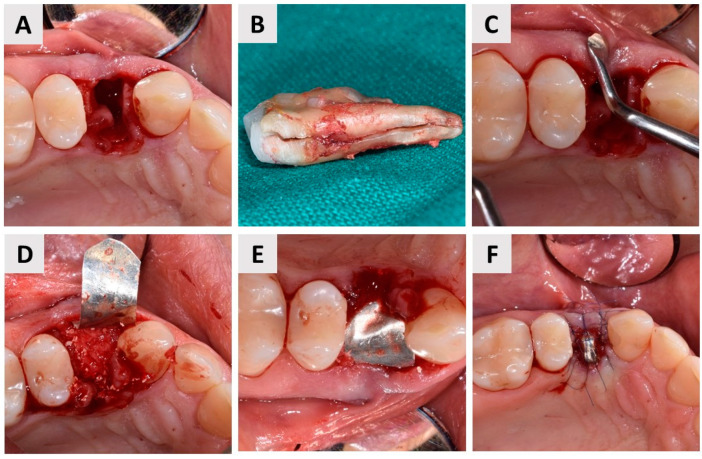
Magnesium membrane shield technique for alveolar ridge preservation (ARP) in complete buccal bone loss (buccal dehiscence); (**A**) Occlusal view of the extraction socket. After administration of local anesthesia, the tooth was extracted as atraumatically as possible. The socket was carefully debrided using a surgical curette, with a finger placed buccally to protect the gingiva, which lacked an underlying bone base; (**B**) The extracted tooth (24, FDI notation) showing a visible vertical root fracture; (**C**) Assessment of the buccal mucosa with an instrument, confirming the absence of the buccal bone wall; (**D**) A gentle elevation of the soft tissue was performed on both the buccal and palatal sides to facilitate the insertion of the magnesium membrane (NOVAMag^®^ membrane, botiss biomaterials GmbH, Berlin, Germany). After the socket was filled with a bovine-derived xenograft (cerabone^®^, botiss biomaterials GmbH, Zossen, Germany), the membrane was positioned between the bone graft material and the soft tissue following the contours of the pre-existing buccal wall; (**E**) The magnesium membrane was then carefully shaped to curve over the grafted area and positioned beneath the palatal gingiva, adjacent to the palatal bone wall; (**F**) Finally, the membrane was stabilized and secured in place with 6-0 sutures (Luxylene, Weiswampach Luxembourg), allowing healing via secondary intention without primary wound closure.

The patient began antibiotic prophylaxis on the day of surgery with Klavocin^®^ (875 mg amoxicillin + 125 mg clavulanic acid, Pliva, Zagreb, Croatia) and continued the same oral antibiotics twice daily, every 12 h, for seven days postoperatively. The procedure was carried out under local infiltration anesthesia (Ubistesin^®^ Forte 40 mg/mL articaine with epinephrine 0.01 mg/mL, 3M Deutschland GmbH, Seefeld, Germany). After atraumatic tooth extraction, alveolar ridge preservation was achieved following the protocol described in Section 2.2. (figures and detailed explanations of the clinical phases—Figure 4). As demonstrated, the magnesium membrane was left exposed to heal via an open healing approach (Figure 4F).

Postoperative instructions included rinsing the oral cavity with a chlorhexidine mouthwash (PerioPlus^®^ 0.2%, Curaprox, Flawil, Switzerland) twice daily for two weeks to reduce bacterial load and aid in healing. Pain management recommendations were provided, advising the use of analgesics as needed. The patient returned for suture removal two weeks post-surgery.

### 2.4. Healing Phase and Dental Implant Placement Planning

**Figure 5:**
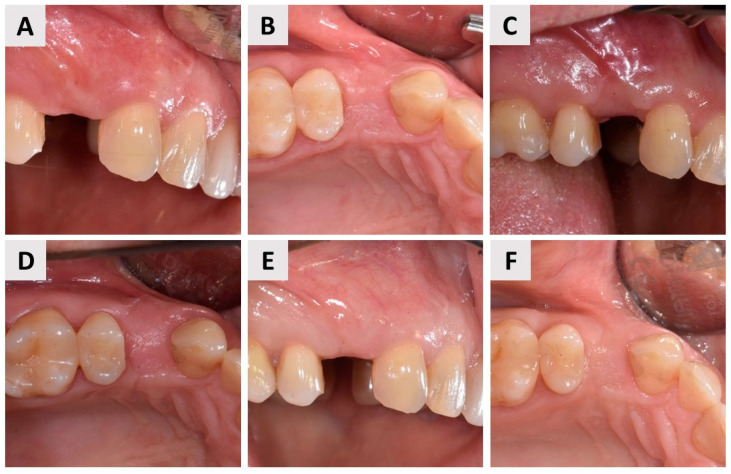
Clinical images of gingival healing at 2, 3, and 6 months post-procedure. (**A**,**B**) Gingival appearance at 2 months post-procedure, shown from the buccal (**A**) and occlusal (**B**) perspectives; (**C**,**D**) Gingival condition at 3 months post-procedure, viewed from the buccal (**C**) and occlusal (**D**) aspects; (**E**,**F**) Gingival status at 6 months post-procedure, depicted from the buccal (**E**) and occlusal (**F**) angles; throughout the healing period, no complications were detected, with the gingival tissues showing steady progress.

**Figure 6:**
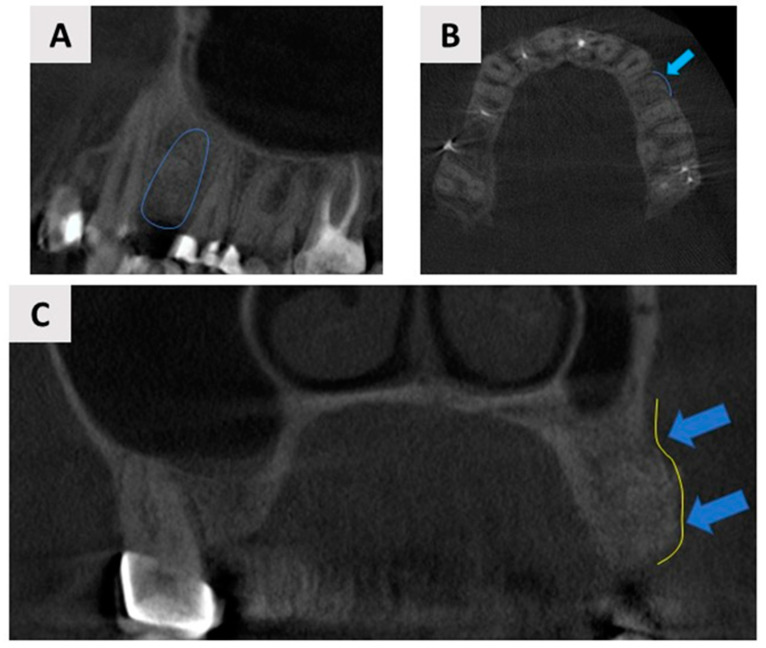
Cone-beam computed tomography (CBCT) assessment of the augmented site 6 months post-surgery. The 3D CBCT scan demonstrated satisfactory bone volume for dental implant placement. (**A**) Panoramic view of the augmented site, with the blue outline indicating the area of regeneration where the dental implant will be placed; (**B**) Axial view showing the regenerated buccal bone wall, highlighted by the blue arrows and outline; (**C**) Coronal plane, with blue arrows and outlines marking the newly regenerated buccal bone wall.

The patient underwent regular follow-up appointments at 2 months (Figure 5A,B), 3 months (Figure 5C,D), and 6 months (Figure 5E,F) postoperatively, during which clinical examinations were conducted to assess the progress of healing. Throughout this period, the healing process was uneventful, with no subjective complaints from the patient or objective signs of complications observed. At each follow-up, the soft tissue condition, graft stability, and overall integration of the magnesium membrane and bone graft material were carefully evaluated. The gingival tissues demonstrated healthy healing. Additionally, radiographic assessments were performed to monitor bone regeneration and confirm adequate bone volume and density for future implant placement (Figure 6).

Based on the clinical and radiological findings, the site demonstrated sufficient bone maturation and soft tissue health, allowing the planning of dental implant placement. The implant placement procedure was scheduled following the completion of the 6-month healing phase to ensure optimal osseointegration conditions.

### 2.5. Dental Implant Placement and Bone Biopsy Harvesting

**Figure 7:**
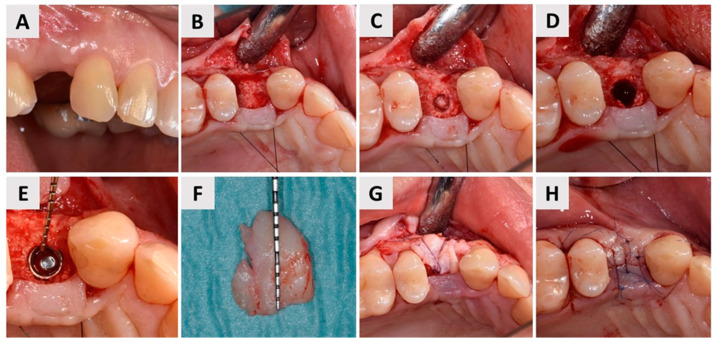
Implant placement and soft tissue augmentation. (**A**) Buccal view of the surgical site at tooth 24 (FDI Notation) after six months of healing. The stable contour of the buccal soft tissue and preserved alveolar ridge are evident; (**B**) Elevation of the full-thickness mucoperiosteal flap, exposing the underlying alveolar bone for implant bed preparation; (**C**) Bone biopsy being obtained using a trephine bur with a 2 mm inner diameter, which was carefully chosen to avoid damage to the implant bed; (**D**) View of the implant bed; (**E**) Placement of the dental implant (4.1 × 10 Straumann^®^ BLT, Basel, Switzerland) into the prepared implant bed. The probe revealed a sufficient buccal bone wall thickness of approximately 3 mm surrounding the implant; (**F**) A free gingival graft, harvested from the maxillary tuberosity and deepithelialized extraorally, was prepared for augmentation to enhance the soft tissue volume around the implant site; (**G**) The tissue graft was secured over the buccal aspect of the implant with 6-0 sutures (Surgicryl^®^ Monofast, St. Vith, Belgium) to enhance tissue thickness and promote optimal healing; (**H**) Primary wound closure was achieved with interrupted 6-0 non-resorbable sutures, ensuring tension-free closure and promoting uneventful healing.

Following the six-month follow-up examination (Figure 7A), the patient underwent dental implant placement. The same preoperative protocol as that used for the previous magnesium membrane shield technique for ARP of the buccal bone wall was followed, including antibiotic prophylaxis with Klavocin^®^ (875 mg amoxicillin + 125 mg clavulanic acid, Pliva, Zagreb, Croatia) and oral rinsing with 0.12% chlorhexidine. A full-thickness mucoperiosteal flap was carefully elevated to expose the surgical site (Figure 7B). Then, as mentioned before, for scientific purposes, the patient consented to a bone biopsy prior to implant placement. To minimize unnecessary bone loss, a trephine bur (Ø 2.0 mm inner diameter, Devmed, Neuhausen ob Eck, Germany) with a smaller diameter than the final implant bed drill was used to harvest a bone tissue sample from the implant site (Figure 7C,D). The bone biopsy was immediately fixed in a 4% formaldehyde solution. Decalcification was performed using Solvagreen (Carl Roth, Karlsruhe, Austria) before the sample was processed in a tissue processor (SLEE MTP, Mainz, Germany) and embedded in paraffin (SLEE MPS/P, Mainz, Germany). Sections of 5 μm thickness were prepared from the paraffin blocks and stained with hematoxylin–eosin. The histological analysis was carried out using a light microscope (Leica DMRB, Leica Microsystems GmbH, Wetzlar, Germany) connected to a video camera (Axio Imager M2, Zeiss, Oberkochen, Germany) for qualitative assessment of the regenerated bone. Subsequently, the dental implant (4.1 × 10 Straumann^®^ BLT, Basel, Switzerland) was placed using a standard implantology set (Figure 7E). A free gingival graft, which was harvested from the maxillary tuberosity and deepithelialized extraorally and then secured over the buccal aspect of the implant with sutures, was used to enhance soft tissue thickness (Figure 7F,G). Primary wound closure was achieved with individual 6-0 non-resorbable sutures (Luxylene, Weiswampach Luxembourg) (Figure 7H). Postoperatively, the patient was instructed to continue oral rinsing with 0.12% chlorhexidine twice daily and prescribed non–steroidal anti-inflammatory drugs as needed for pain management.

## 3. Results

### 3.1. Clinical Outcomes

**Figure 8:**
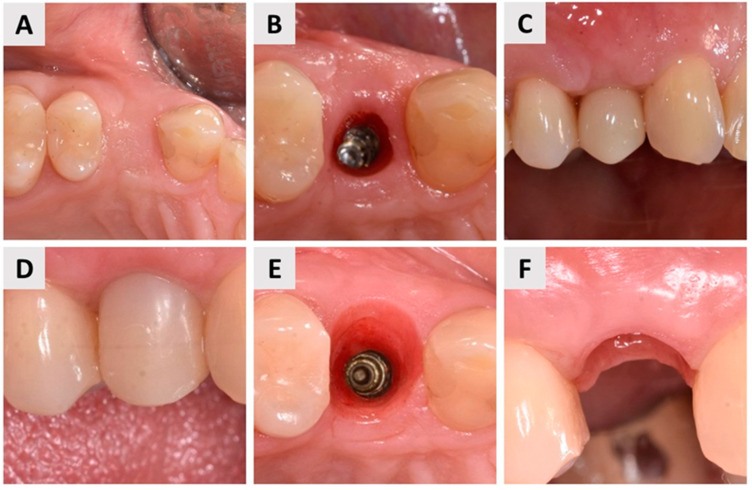
Optimization of soft tissue health and contouring. (**A**) Occlusal view of the healed implant site four months after dental implant placement and soft tissue grafting, showing stable soft tissue contours; (**B**) Occlusal view of the dental implant exposure, marking the beginning of the prosthetic phase; (**C**) Initial stage of soft tissue conditioning with a provisional crown placed to guide tissue adaptation; (**D**) Provisional crown in place after three months, allowing soft tissue maturation at the implant site; (**E**) Occlusal view of the implant site after three months of soft tissue contouring with the provisional crown, demonstrating significant improvement in soft tissue contours compared to the initial exposure (compared with (**B**)); (**F**) Final emergence profile, buccal view, showing optimal soft tissue health and contouring and thus preparedness for definitive prosthetic restoration.

Four months after soft tissue grafting and dental implant placement, the patient returned to the dental office to initiate the definitive implant-prosthetic restoration. Clinical examination revealed well-maintained and stable soft tissue contours, indicating successful healing (Figure 8A). The dental implant was exposed (Figure 8B), and the next phase focused on optimizing soft tissue health and contouring. To achieve this, a provisional crown was fabricated and placed to guide soft tissue conditioning, ensuring an ideal emergence profile. The provisional crown remained in situ for a total of three months (Figure 8C), during which time the soft tissues adapted and developed a stable, individualized emergence profile (Figure 8D,E).

**Figure 9:**
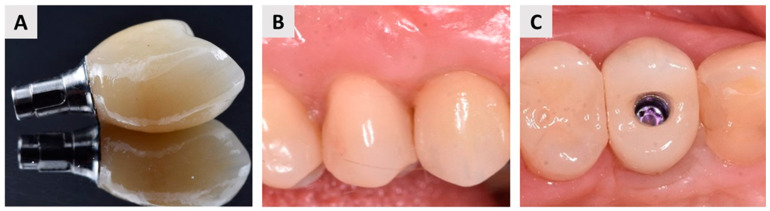
Final prosthetic restoration. (**A**) Definitive zirconia ceramic crown; (**B**) Facial (buccal) view of the final prosthetic restoration, highlighting the harmonious integration with the surrounding soft tissues, a precise fit, and aesthetic quality; (**C**) Occlusal view of the final restoration, demonstrating the optimal contour and alignment of the zirconia crown with the adjacent dentition, as well an optimal buccal bone wall profile.

Following successful tissue shaping, impressions were taken, and the final prosthetic restoration was prepared. A zirconia ceramic crown was fabricated and secured onto the implant abutment using a screw-retained method (Figure 9). This final restoration provided both excellent esthetics and functional outcomes, with the patient expressing satisfaction with the result.

### 3.2. Histological Outcomes

**Figure 10:**
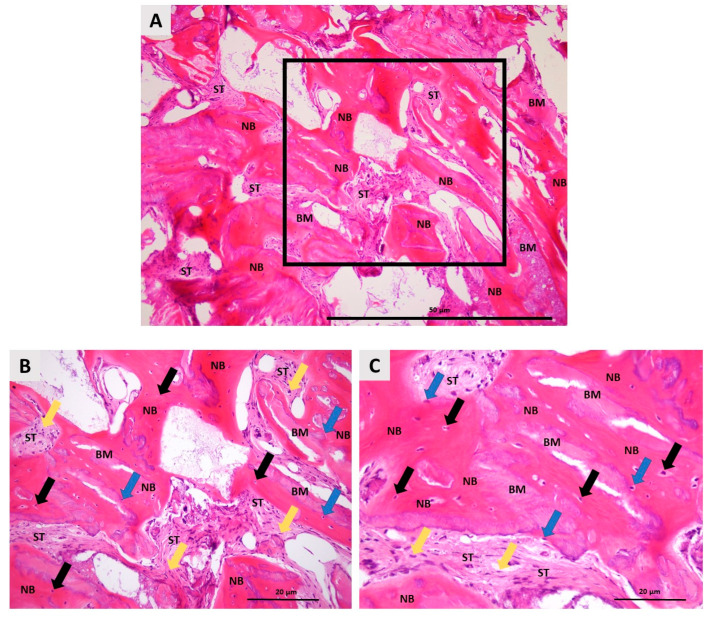
Histologic photomicrographs of the bone biopsy taken six months after ARP using the magnesium membrane shield technique. (**A**) A photomicrograph shows newly formed bone (NB) in direct contact with residual biomaterial (BM), with no signs of inflammation, indicating successful integration of the bone graft. Between the newly formed bone and the biomaterial aggregates is soft tissue (ST). The highlighted square marks the area that was further examined at higher magnification (hematoxylin–eosin stain, 100× magnification); (**B**) A higher magnification view shows details such as osteocytes (black arrows) embedded in the calcified bone matrix, confirming maturation of the newly formed bone. The surrounding soft tissue (ST) is composed mostly of fibroblast cells (yellow arrows). Osteoblasts (blue arrow) on the bone surface indicate ongoing bone remodeling and dynamic osteogenesis (hematoxylin–eosin stain, 200× magnification); (**C**) A detailed photomicrograph focusing on the interaction between the newly formed bone (NB) and the remaining biomaterial (BM), showing the tight integration of these structures (hematoxylin–eosin stain, 400× magnification).

Histological analysis of the bone biopsy taken six months after the magnesium membrane shield technique for ARP revealed a well-organized, homogeneous bone structure with no signs of inflammation. The newly formed bone was in close contact with the remaining bovine xenograft, indicating successful integration. Osteocytes, the mature bone cells, were clearly visible within the lacunae, indicating that the bone matrix was mature. In addition, active osteoblasts were observed along the interface between the newly formed bone and the biomaterial, confirming ongoing bone formation. The surrounding soft tissue showed a dense population of fibroblasts, further supporting tissue regeneration and stability at the augmentation site (Figure 10). These results indicate successful osteoconduction and biocompatibility of the magnesium membrane in promoting bone regeneration.

Histomorphometric evaluation revealed 47% newly formed bone, 32% soft tissue, and 19% residual biomaterial.

## 4. Discussion

Various modalities of ARP techniques have been documented in the literature, which can be categorized into three primary approaches: socket grafting using different types of biomaterials, sealing the orifice of the extraction socket with autogenous or substitute tissues, and a combination of both techniques, employing either primary or secondary intention healing [28,29,30]. Currently, there is no conclusive evidence to support the superiority of any one modality over the others within the spectrum of ARP techniques [17,28]. In this representative step-by-step case, the magnesium membrane shield technique combined with bone grafting was used to restore the buccal bone wall. This not only contributed to functional and aesthetic outcomes but was also confirmed through histological analysis, which further validated the tissue regeneration process.

Magnesium has been successfully used in both cardiovascular [31,32,33] and orthopedic procedures [34,35,36,37] due to its biocompatibility and favorable resorption profile. This stability, combined with gradual resorption, means that the magnesium membrane initially retains its shape to allow regeneration without the need for a second procedure to remove it. These beneficial properties are now being effectively incorporated into dental medicine, positioning the magnesium membrane as an innovative advancement in bone regeneration. As a resorbable metal membrane, it offers exceptional mechanical strength during the healing phase, eliminating the need for a second surgical procedure for removal [25,38]. Its adaptability has been demonstrated across various applications in vivo, including horizontal and vertical ridge augmentation [24] and sinus lift procedures [23], with excellent outcomes. The use of a magnesium membrane effectively avoids complications such as membrane collapse, as it maintains its mechanical integrity throughout the healing period. An in vivo study by Rider et al. [25] on the corrosion kinetics of magnesium membranes demonstrated that during the degradation process, magnesium salts are formed. These salts preserve the structural framework of the membrane, allowing it to retain its original form during the critical healing phases at 1, 2, and 4 weeks. This property ensures continued mechanical support while facilitating tissue regeneration. In addition, magnesium membranes have shown beneficial biological properties that are supported by both in vitro and in vivo studies [39,40,41,42]. Magnesium ions released during the degradation of the membrane have been shown to facilitate soft tissue adhesion, particularly promoting the proliferation of gingival fibroblasts, which are essential for effective wound healing [41,43]. Also, magnesium ions play a critical role in enhancing bone development by promoting the attachment, proliferation, and differentiation of osteoblasts, which are essential for bone healing. Specifically, magnesium ions have been shown to accelerate mineralization by binding to Type I collagen, facilitating the expression of osteogenic-related factors [44]. The magnesium-rich environment created during degradation activates signaling pathways involved in osteogenesis, supporting calcium deposition and bone formation [45,46]. Overall, this biological activity contributes to the magnesium membrane’s ability to promote and ensure a stable environment for both soft and hard tissue regeneration.

Magnesium membranes, like collagen membranes, are fully resorbable, which distinguishes them from titanium meshes, which are non-resorbable and contain pores that allow vascularization. While these pores are advantageous in promoting vascularization, they can compromise the barrier function of the titanium mesh, allowing soft tissue to infiltrate the defect area, which may interfere with optimal bone regeneration within the defect site [47,48]. From that aspect, the magnesium membrane combines the benefits of titanium and collagen membranes and offers rigidity with full resorbability. Studies have shown that the degradation process of the magnesium membrane releases magnesium salts that actively attract blood vessels and osteoblasts to the site of regeneration. This increased vascularization contributes to denser, more robust bone formation and offers a significant advantage over titanium in bone regeneration [41,49,50,51,52].

In this case, we highlight yet another successful application of the magnesium membrane in a highly demanding scenario, both aesthetically and functionally. Namely, teeth extracted in clinical practice often present with a poor prognosis due to advanced periodontal disease or various pathological processes involving the root. These conditions frequently result in significant bone loss, particularly in the buccal wall, which complicates alveolar ridge preservation and subsequent implant placement [53,54]. Based on clinical and radiological assessments, with further confirmation by direct visual inspection after tooth extraction, the socket was classified as defect type ST3C according to the classification of Steigmann et al. [27]. This type of defect is characterized by buccal bone dehiscence exceeding two-thirds of the wall height. Steigmann et al. recommend flap elevation, placement of a non-resorbable d-PTFE membrane over the bone graft material, and fixation with a cross suture to treat such defects. The membrane is usually left in place for 4–6 weeks to allow physiologic bone maturation. However, the use of a d-PTFE membrane requires a second surgical procedure to remove it [55,56]. In contrast, the magnesium membrane used in this case offers significant advantages due to its complete resorbability, which, from a clinical aspect, simplified the treatment protocol and improved patient comfort. An additional benefit of this technique is demonstrated in Figure 4F, which shows that the membrane was left exposed, facilitating healing via an open approach. This approach eliminates the need for a full mucoperiosteal flap, including periosteal incisions and mucogingival dislocation, which significantly reduces surgical trauma and preserves the existing soft tissue architecture [5,57,58]. The open healing method not only enhances postoperative monitoring, enabling early detection of complications and fostering a favorable wound healing environment, but also presents three key advantages: it avoids mucogingival border dislocation, results in a broader band of keratinized mucosa after healing, and reduces the postoperative burden on the patient, with fewer reports of swelling and pain [5,59]. In our case, it was clearly evident that in areas where the membrane was exposed, secondary intention healing occurred, leading to the formation of healthy, keratinized mucosa (Figure 7 and Figure 8).

Any fixation system can be used to fix magnesium membranes [23,24,38]. In this case, however, such use was unnecessary for two reasons. First, screws or pins are usually required to stabilize resorbable membranes due to their lack of structural rigidity [60,61]. Namely, resorbable membranes like collagen rely on fixation systems, tenting screws, or the underlying graft for stability [62,63]. The dimensionally stable structure of the magnesium membrane, however, simplifies the procedure and enhances its suitability for guided bone regeneration applications, and its rigidity is not dependent on the underlying graft or additional fixation. In addition, the stiffness of the magnesium membrane in this case report allowed insertion without complete elevation of the mucoperiosteal flap (Figure 4), which is another benefit of this surgical approach, as previously described. However, its physical properties have certain limitations, as magnesium begins to disintegrate immediately upon contact with oxygen and body fluids and requires precise handling to prevent premature resorption [20].

Our surgical approach has similarities with the technique described by Elad et al. [23] in their case series, which also used bone grafting in combination with magnesium membranes as a shield for ridge preservation. However, there are some differences in approach, as in our case, the magnesium membrane shield technique was used with an open healing approach and delayed implant placement, whereas Elad et al. used immediate implant placement, a protocol that typically requires a different healing dynamic. This different timing provides a perspective on the ability of the magnesium membrane to promote bone regeneration without the immediate mechanical stress of an implant, which could expand its potential application in more complex cases of ridge preservation.

Bone healing is a dynamic process that occurs in three distinct phases: (1) the coagulum phase, (2) the inflammation phase, and (3) the proliferation phase [59]. A critical factor for successful wound healing, particularly in the context of open healing, is that the membrane used must support the healing environment without causing any adverse effects [64]. As mentioned before, the magnesium membrane fulfills this requirement by being easily degraded and absorbed in physiological conditions, thereby creating a magnesium-rich environment that activates various signaling pathways and promotes calcium deposition and osteogenesis [58]. During the transition from the inflammatory phase to the proliferative phase, the pH of the surrounding tissue shifts from acidic to alkaline. This change is essential for proper bone regeneration [65,66]. The degradation of the magnesium membrane contributes to this process by creating an alkaline environment in the immediate vicinity of the membrane, which not only supports osteogenesis but also reduces the likelihood of inflammation [25]. The alkaline pH resulting from magnesium membrane degradation may therefore potentially contribute to facilitating the transition from the inflammation phase to the proliferation phase.

Bacterial colonization is another significant concern in ARP procedures. Namely, bacterial colonization on the surface of collagen membranes can accelerate their degradation due to the activity of collagenase, an enzyme produced by bacteria that degrades collagen [67]. In contrast, microorganisms cannot degrade magnesium membranes through enzymatic action as they do with collagen membranes. Additionally, magnesium membranes possess inherent antibacterial properties, which further reduce the risk of bacterial infection affecting the underlying bone graft [68,69]. This characteristic was particularly advantageous in our case for two key reasons: first, in the open healing approach, a portion of the bone defect, graft, and magnesium membrane remains exposed to the oral cavity environment—a setting conducive to bacterial growth due to moisture, temperature, and the presence of food residues. Second, the vertical fracture of the tooth prior to extraction likely resulted in bacterial colonization within the fracture line. Despite the extraction, some bacteria may remain in the bone defect, posing a risk of graft infection. The antibacterial properties of the magnesium membrane serve to minimize this risk, providing an added layer of protection to the graft and ensuring a more favorable healing environment.

Although this case report demonstrates the successful application of the magnesium membrane shield technique for preserving the alveolar ridge in cases of severe buccal bone wall destruction, it remains a preliminary observation with limitations typical of single-case reports. The results cannot be generalized, and there is a risk of over-interpretation without evidence of a clear cause-effect relationship. Further studies, particularly randomized clinical trials, are needed to validate the technique’s efficacy, safety, and long-term outcomes, as well as to evaluate its potential in long-span defects involving multiple extraction sites. Despite these limitations, this report represents the first documented use of the magnesium membrane shield technique in cases of severe buccal bone deficiency. The promising results observed, including effective bone regeneration and soft tissue preservation, suggest that the magnesium membrane may be a viable solution for managing challenging ridge preservation. Future studies should aim to confirm these findings and further explore the potential of this novel approach for advanced bone regeneration.

## Data Availability

The data presented in this article are available upon request from the corresponding author.

## Supplementary Materials

The following supporting information can be downloaded at: https://www.mdpi.com/article/10.3390/biomedicines12112537/s1, File S1: CARE Checklist of information to include when writing a case report.
