# Supplementary material for: Magnesium Membrane Shield Technique for Alveolar Ridge Preservation: Step-by-Step Representative Case Report of Buccal Bone Wall Dehiscence with Clinical and Histological Evaluations
Source: Biomedicines. 2024 Nov 6;12(11):2537. doi: 10.3390/biomedicines12112537 (PMC11591876; doi:10.3390/biomedicines12112537)
Supplement: Supplementary file 1 [file biomedicines-12-02537-s001.zip › biomedicines-3296623-supplementary-File S1.pdf]

CARE case report guidelines

CARE Checklist of information to include when writing a case report

CC BY-NC-ND

Topic	Item	Checklist item description	Reported on Line
**Title**	1	The diagnosis or intervention of primary focus followed by the words "case report"	yes, lines 1 – 4
**Key Words**	2	2 to 5 key words that identify diagnoses or interventions in this case report, including "case report"	yes, line 50
**Abstract (no references)**	3a	Introduction: What is unique about this case and what does it add to the scientific literature?	yes, lines 28 - 31
	3b	Main symptoms and/or important clinical findings	yes, lines 34 - 35
	3c	The main diagnoses, therapeutic interventions, and outcomes	yes, lines 34 - 38
	3d	Conclusion—What is the main "take-away" lesson(s) from this case?	yes, lines 46 - 49
**Introduction**	4	One or two paragraphs summarizing why this case is unique (**may include references**)	yes, lines 97 - 101
**Patient Information**	5a	De-identified patient specific information	yes, 112 - 119
	5b	Primary concerns and symptoms of the patient	yes, 112 - 119
	5c	Medical, family, and psycho-social history including relevant genetic information	yes, 113 - 119
	5d	Relevant past interventions with outcomes	yes, 115 -117
**Clinical Findings**	6	Describe significant physical examination (PE) and important clinical findings	yes, 115 - 119
**Timeline**	7	Historical and current information from this episode of care organized as a timeline	yes, 112 - 119
**Diagnostic Assessment**	8a	Diagnostic testing (such as PE, laboratory testing, imaging, surveys).	yes, 115 - 119
	8b	Diagnostic challenges (such as access to testing, financial, or cultural)	non-applicable
	8c	Diagnosis (including other diagnoses considered)	yes, 124 - 125
	8d	Prognosis (such as staging in oncology) where applicable	non-applicable
**Therapeutic Intervention**	9a	Types of therapeutic intervention (such as pharmacologic, surgical, preventive, self-care)	yes, 131 - 169
	9b	Administration of therapeutic intervention (such as dosage, strength, duration)	yes, 146 – 147, 148 – 151, 171 – 172, 180 – 181, 229 - 231
**Follow-up and Outcomes**	9c	Changes in therapeutic intervention (with rationale)	non-applicable
	10a	Clinician and patient-assessed outcomes (if available)	yes, 119 – 225 and 265 – 295
	10b	Important follow-up diagnostic and other test results	yes, 200 – 212
	10c	Intervention adherence and tolerability (How was this assessed?)	non – applicable
	10d	Adverse and unanticipated events	non – applicable (no complications were reported)

**Discussion**	**11a**	A scientific discussion of the strengths AND limitations associated with this case report	yes, 431 - 443
	**11b**	Discussion of the relevant medical literature **with references**	yes, 334 - 340
	**11c**	The scientific rationale for any conclusions (including assessment of possible causes)	yes, 438 - 443
	**11d**	The primary "take-away" lessons of this case report (without references) in a one paragraph conclusion	yes, 438 - 443
**Patient Perspective**	**12**	The patient should share their perspective in one to two paragraphs on the treatment(s) they received	yes, line 288 - 290
**Informed Consent**	**13**	Did the patient give informed consent? Please provide if requested	**Yes** ■ **No** ☐
